# Spiropyran Meets Guanine Quadruplexes: Isomerization Mechanism and DNA Binding Modes of Quinolizidine‐Substituted Spiropyran Probes

**DOI:** 10.1002/chem.202001586

**Published:** 2020-09-17

**Authors:** Davide Avagliano, Pedro A. Sánchez‐Murcia, Leticia González

**Affiliations:** ^1^ Institute of Theoretical Chemistry Faculty of Chemistry University of Vienna Währinger Straße 17 1090 Vienna Austria; ^2^ Vienna Research Platform on Accelerating Photoreaction Discovery University of Vienna Währinger Straße 17-A 1090 Vienna Austria

**Keywords:** guanine-quadruplexes, QM/MM, spiropyrans, umbrella sampling

## Abstract

The recent delivery of a fluorescent quinolizidine‐substituted spiropyran, which is able to switch in vivo and bind to guanine quadruplexes (G4) at physiological pH values, urged us to elucidate its molecular switching and binding mechanism. Combining multiscale dynamical studies and accurate quantum chemical calculations, we show that, both in water and in the G4 environment, the switching of the spiropyran ring is not promoted by an initial protonation event—as expected by the effect of low pH solutions—but that the deprotonated merocyanine form is an intermediate of the reaction leading to the protonated open species. Additionally, we investigate the binding of both deprotonated and protonated open forms of merocyanine to c‐MYC G4s. Both species bind to G4s albeit with different hydrogen‐bond patterns and provide distinct rotamers around the exocyclic double bond of the merocyanine forms. Altogether, our study sheds light on the pharmacophoric points for the binding of these probes to DNA, and thereby, contributes to future developments of new G4 binders of the remarkable family of quinolizidine‐substituted spiropyrans.

## Graphical abstract

**Protonate me, if you can**: Multiscale calculations show that the opening of the fluorescent quinolizidine‐substituted spiropyran is not proton‐assisted and that the deprotonated merocyanine open form is an intermediate, both in solution and in guanine quadruplexes.

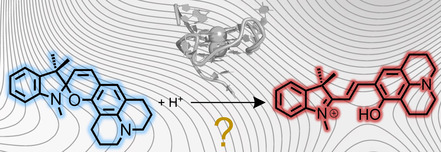

## Introduction

Guanine quadruplexes (G4s) are non‐canonical secondary structures that can be adopted by particular guanine‐rich sequences.^1^ They are constituted by stacked guanine tetrads (G‐tetrads), formed by four guanine bases interacting with each other through Hoogsteen hydrogen bonding, and chelating a central metal cation. Depending on the relative orientation of the phosphate backbone, strand direction, and specific base‐sequence, these guanine‐reach RNA or DNA can fold following different patterns and resulting in a wide diversity of topological families of G4s.^2^, ^3^ G4s have been found to be abundant in cancer‐related genes as well as in the telomeres of the chromosomes, and in recent years they have emerged as promising therapeutic targets to silence oncogenes.^4^ For these reasons, it is important to identify new probes that bind, stabilize, probe, or damage G4s.^5^, ^6^

**Scheme 1:**
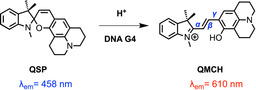
Quinolizidine‐substituted spiropyrans (QSP) isomerizes to the protonated merocyanine (QMCH) under acid conditions or by DNA G4 binding. Bonds for the definition of the QMCH isomers are shown in blue (α, β, γ).^9^

Quinolizidine‐substituted spiropyran (QSP, see Scheme 1) is a fascinating example of an organic fluorescent dye with the ability to specifically target G4s over single‐ and double‐stranded DNA. QSP can change its fluorescence emission maximum spatiotemporally, switching from its closed spiropyran form to the open protonated merocyanine form (QMCH) in acid media (e.g., inside a lysosome) or upon binding in vivo to G4s of regulatory genes such as c‐MYC under physiological pH.^7^ In the QMCH form, the emission is redshifted to 610 nm compared with the QSP form (emission at 458 nm). The evidence that QSP opens to QMCH in acid environment as well as in the presence of c‐MYC G4 (at neutral pH values), suggests that the conversion from QSP to QMCH is a proton‐mediated process. Although other spiropyran derivatives have been observed to isomerize in the presence of acid as well, they require more severe conditions (e.g., trifluoroacetic acid):^8^ indoline benzospiropyran (BIPS) derivatives present lower p*K*_a_ values than QSP (p*K*_a_≈5.9) making pH‐mediated opening under physiological conditions inviable. Thus, quinolizidine‐spiropyrans constitute a unique family of probes to be used in vivo.

**Scheme 2:**
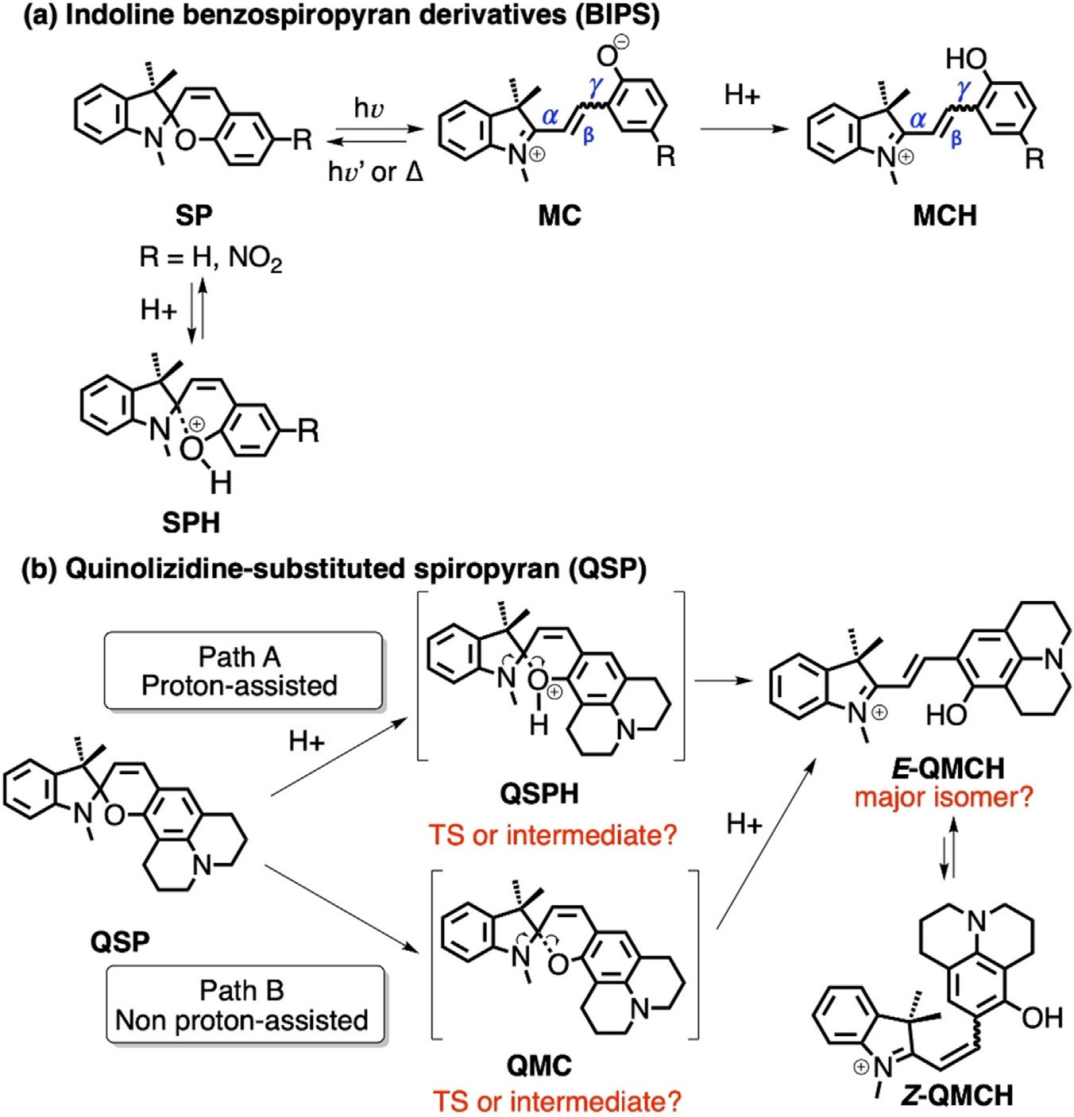
(a) The protonation of BIPS SP prevents SP from evolving to MCH. (b) Two possible reaction mechanisms for the QSP–QMCH switching process in solutions with low pH values.

The protonation mechanism of SPs has been extensively discussed. Wojtyk and co‐workers^8^ employed ^1^H NMR and UV/Vis spectroscopy to show that the protonated species of 6‐nitro BIPS is spontaneously generated upon protonation of the oxygen in the presence of increasing concentrations of acid (Scheme 2 a). The authors proposed that the SPH species acts as an ‘unreactive sink’, trapping 6‐nitro BIPS SP and competing with the SP→MCH opening process. The addition of an extra base to SPH restores the concentration of SP, and in the presence of proper light illumination, promotes the switching to the open MC/MCH species. More recently, Browne and co‐workers^10^ showed that under controlled pH conditions and UV irradiation both, MC and MCH *E*‐isomers of unsubstituted and 6‐nitro BIPS can be formed in solution. Nevertheless, the access to the *E*‐isomer of the MCH species required the UV illumination of SP for at least one of the steps. It must be stressed that the *E*‐isomer of the exocyclic double bond in MCH (β‐bond in Scheme 2 a) can present an ensemble of rotamers owing to the relative orientation of the two rings with respect to the exocyclic double bond (α and γ bonds).^9^ The TTC isomer (*E*‐*E*‐*Z* for α, β, and γ bonds, respectively) has been reported to be the most stable form of both MC and MCH species.^11^

In contrast to the former BIPS derivatives, QSP points to a different switching mechanism where no light is required and where protonation seems to be involved. However, no details in that regard—except for some initial structure‐based design campaign^7^—are known owing to the absence of theoretical calculations. As shown in Scheme 2 b, the ring opening from QSP to QMCH requires the breaking of the sigma bond C_spiro_−O and the protonation of this oxygen. Thus, depending on the order of these two processes, there will be at least two possible reaction mechanisms (paths A and B, Scheme 2 b). The aim of the present work is twofold: first, we identify the most probable chemical pathway to activate QSP in solution at low pH values and the nature of its intermediate species. Subsequently, we provide an atomistic molecular model for the binding mode of QSP to the parallel‐stranded G4 of the c‐MYC promoter,^12^ beyond static molecular docking studies,^7^ by using extensive molecular dynamics simulations up to the μs‐scale. To fulfil these aims, we employ state‐of‐the‐art complementary approaches including quantum mechanical (QM), mixed quantum and classical molecular mechanical (QM/MM), and molecular dynamics (MD) simulations. The results provide a comprehensive picture of QSP chemistry, from solution to its binding to G4 upon thermal opening.

## Results and Discussion

First of all, we investigated the two possible reaction mechanisms leading to the formation of QMCH from QSP (recall Scheme 2 b). In path A, the protonation of QSP occurs first, with the formation of either a QSPH intermediate or a transition state (TS), before QMCH is generated. In path B, the QSP ring opening and formation of QMC is followed by the subsequent protonation. In contrast to A, in B protonation would not affect the kinetics of the QSP opening, but the thermodynamics of the process.

**Figure 1:**
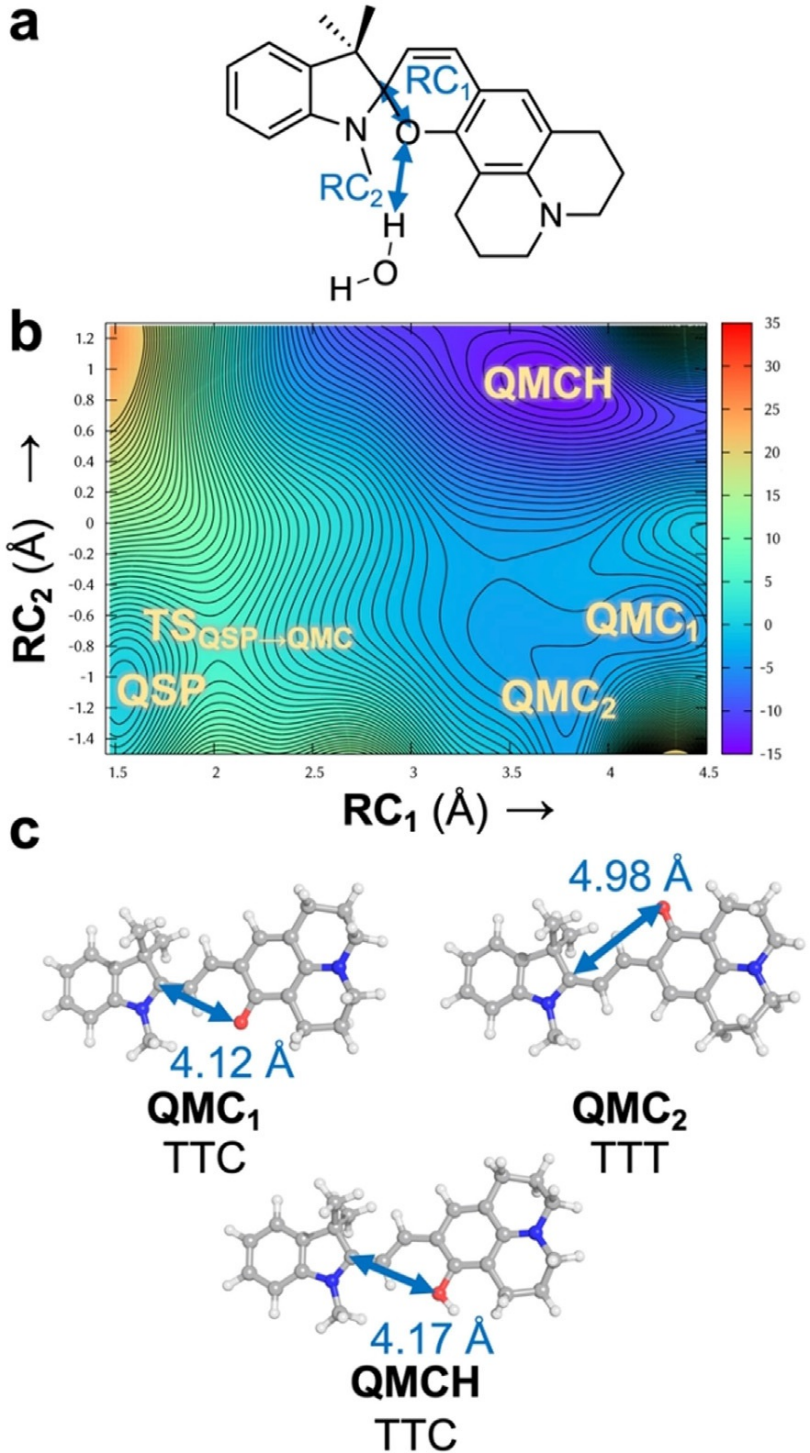
(a) Definition of the reaction coordinates RC_1_ and RC_2_ (Å) as a distance and a linear combination of distances, respectively. (b) 2D free energy surface (kcal mol^−1^) for the reaction QSP→QMC/QMCH computed by means of 2D steered and umbrella sampling QM/MM MD simulations in explicit aqueous solvent. The zero energy is set to the QSP minimum and each contour line represents a difference of 0.5 kcal mol^−1^. (c) Geometries of the local minima identified in the 2D‐US energy surface after optimization in implicit solvent (RI‐MP2/def2‐TZVP@COSMO). The distance for RC_1_ is highlighted with a two‐headed arrow. TTC and TTT differ in the *cis*↔*trans* rotation with respect to the γ bond.

The reaction mechanism was investigated by using steered QM/MM MD simulations together with two‐dimensional umbrella sampling (2D‐US) calculations, both in explicit water solution and bound to c‐MYC G4 (see computational details in Sections S1–S3 of the Supporting Information). These simulations allowed us to (i) observe the C_spiro_−O bond breaking, (ii) explicitly include the effect of the water or DNA environment in the reaction, and (iii) explore two reaction coordinates independently. The systematic exploration of the potential energy surfaces provided stationary points and the minimum free energy pathway. The two reaction coordinates are defined as the bond breaking between the carbon C_spiro_ and the oxygen O atoms of the spiro‐junction (RC_1_) and the proton transfer to O from a water molecule (H_water_) of the solvent (RC_2_; see Figure 1 a). Although Whereas RC_1_ is defined as a single distance between two atoms, RC_2_ is specified as a linear combination of O_water_−H_water_ and O_spiro_−H_water_ distances. In the QM/MM calculations, the QM region—treated with the Density Functional Tight‐Binding (DFTB3) semiempirical level of theory^13^—contains the probe and a water molecule.

Figure 1 b shows the 2D‐US free energy profile of the reaction in water, where 600 windows were required to cover the full 2D free energy space (Figures S1 and S2 in the Supporting Information). Through these simulations, four local minima were identified: the initial QSP, two unprotonated open forms of QMC (QMC_1_ and QMC_2_), and the product QMCH (Figure 1 c). The QMCH species is the thermodynamically most stable and, hence, the formation of a previous protonated QSPH species is unfeasible, neither as a TS nor as an intermediate. In contrast, the two minima, corresponding to the unprotonated QMC_1_ and QMC_2_ intermediates, are thermally accessible through a transition state TS_QSP‐QMC_ lying at 5 kcal mol^−1^. Importantly, once the system reaches the transition state, it can evolve either to the QMC metastable intermediates or directly to the QMCH species. This means that the pH of the aqueous solution can determine the chemical pathway to get QMCH. These results suggest, therefore, that path B is more probable in solution than path A, as no initial protonation of the QSP species is necessary to reach QMCH.

**Scheme 3:**
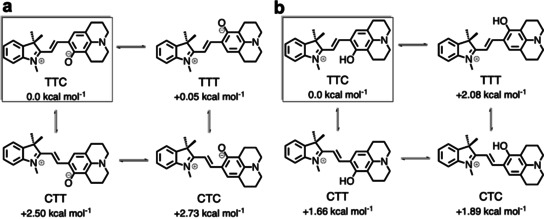
Relative stability of stable rotamers of the *cis*‐*trans E*‐isomer with respect to the α/β bond (TTC, TTT, CTT, CTC) of QMC (a) and QMCH (b) species calculated at RI‐MP2/def2‐TZVP@COSMO level of theory. The zero of the Gibbs energy (kcal mol^−1^, 300 K) is set to the most stable isomer TTC.

The two QMC isomers (QMC_1_ and QMC_2_) are almost degenerate in energy (≈4 kcal mol^−1^) in agreement with the fact that the merocyanine species can adopt different isomers with respect to the double bond (β‐bond). The two obtained rotamers correspond to the *E*‐isomer of the double bond and differ in their relative orientation of the phenyloxy group with respect to the nitrogen of the indoline (i.e., rotation around the α‐bond). To obtain an accurate estimation of the energetics of all stable geometries, four structures, corresponding to possible combinations of rotations around the α/γ‐bonds, were optimized at a higher level of theory (Møller–Plesset second‐order perturbation theory, using the resolution of identity approximation and a large basis set, RI‐MP2/def2‐TZVP, Figure 1 c, Scheme 3, and Supporting Information) with an implicit description of the aqueous solution (the conductor‐like screening COSMO method,^14^ see Section S4 in the Supporting Information). Only *E*‐isomers were explored as no *Z*‐species were found in our 2D‐US studies and the *E*‐isomers have been shown experimentally to be thermodynamically more stable for benzoindolic spiropyrans.^8^

Among the four possible QMC conformations, the TTC and TTT isomers (corresponding to the QMC_1_ and QMC_2_ geometries obtained in the 2D free energy surface, respectively, Figure 1 c) are the energetically lowest minima, with an energy difference of only 0.05 kcal mol^−1^ between them. Thus, both TTC and TTT isomers are equally stable in water. The computed relative energies agree with the DFT results for the 6‐nitro BIPS derivatives. In addition, we also computed the relative energy of the *E*‐isomers of QMCH. In this case, the protonation of the phenol oxygen changes slightly the relative stability, resulting in the TTC isomer being 2.08 kcal mol^−1^ lower in energy than TTT (Scheme 3 b). Our RI‐MP2 calculations agree well with previous experimental data^7^, ^10^ and with the results of the 2D‐US simulation. In summary, QSP would populate—based on the thermodynamics—mainly the TTC isomer of QMC(H) in solution and the protonation would not promote the thermal ring opening.

We then investigated what happens in the G4 context: does the polynucleotide change the reaction mechanism? To answer this question, we studied the reaction in the local environment of G4 with the same methodology. As no structure of QSP bound to G4 is available, we superimposed QSP with the ligand position of a quindoline/c‐MYC G4 complex (PDB id: 2L7V)^12^ and relaxed the whole system with the MD simulation protocol described in Section S1 (in the Supporting Information). As in solution, the probe and the water molecule closest to the oxygen of the spiropyran were considered in the QM region and the same definition for the reaction coordinates RC_1_ and RC_2_ was used, as well as the same number of windows (600) and data points (1000 per window, 600 000 total; see Section S3 in the Supporting Information).

**Figure 2:**
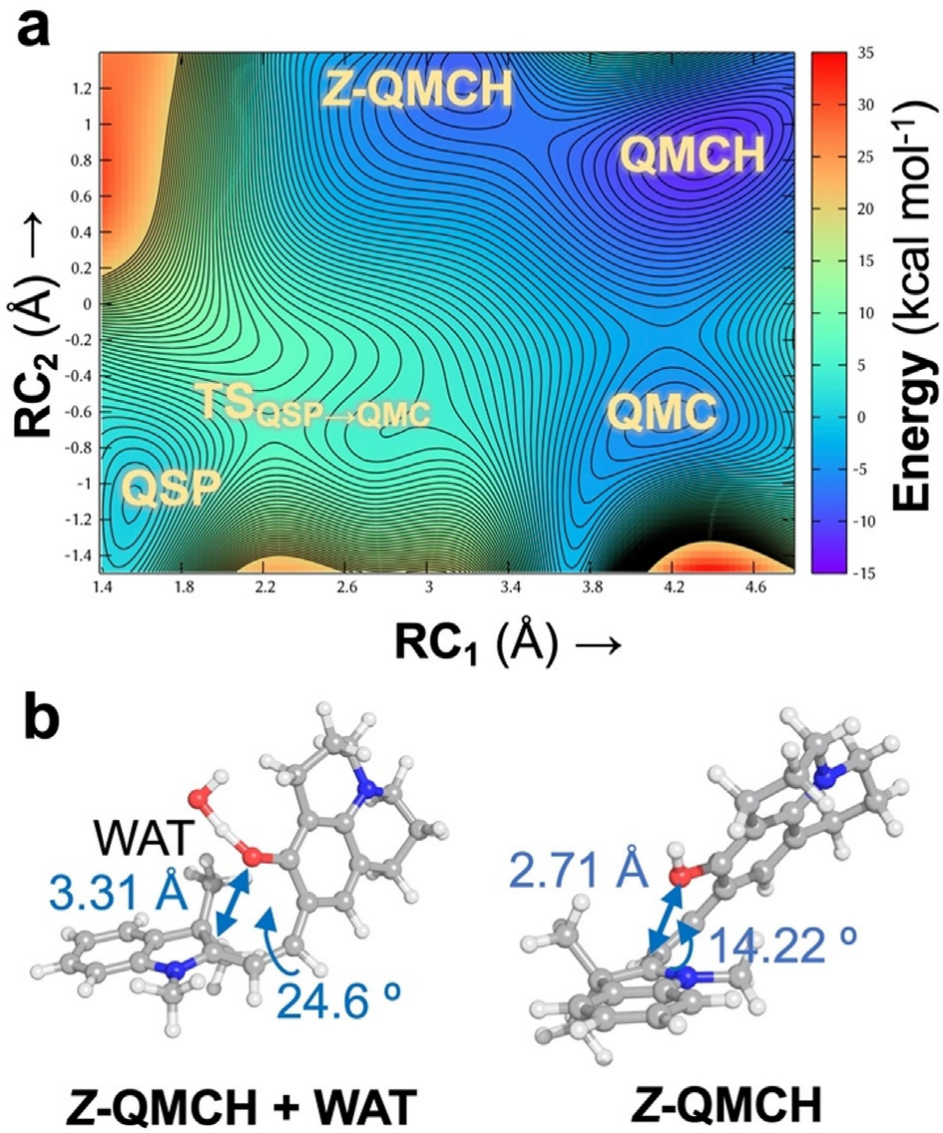
(a) Two‐dimensional free energy surface (kcal mol^−1^) for the opening QSP→QMC/QMCH when bound to G4. The zero energy is set to the QSP minimum and each contour line represents a difference of 0.5 kcal mol^−1^. (b) Identified geometry (*Z*‐MCH+WAT) and optimized geometry of the *Z*‐MCH intermediate at RI‐MP2/def2‐TZVP level of theory in the gas phase.

The obtained 2D free energy surface is shown in Figure 2 a, along its predicted stationary points for QSP, QMC, and QMCH. Accordingly, the pathway leading to QMCH is presumably the same as in water (path B of Scheme 1). The energy barrier of TS_QSP‐QMC_, as well as the relative energies of the QMCH and QMC minima are comparable to those in water. However, in the presence of G4, only the TTC isomer of the QMC form is identified. Further, a new local minimum, *Z*‐QMCH (RC_1_=3.1 Å, RC_2_=1.2 Å) is found in the 2D free energy surface (Figure 2 a). This isomer corresponds to the *Z*‐isomer identified previously by Browne et al.^10^ for the 6‐nitro BIPS (SPH, Scheme 2 a), where the oxygen of the spiropyran is protonated and the C_spiro_−O bond broken. In the gas‐phase optimized geometry (RI‐MP2/def2‐TZVP), the distance between the C_spiro_ and the O atoms is 2.71 Å. In this geometry, the two rings around the exocyclic double bond (β‐bond) are not co‐planar, with a dihedral angle of 14°. The steric repulsion of the two methyl groups on C3 of the indoline ring with the oxygen atom of *Z*‐QMCH prevents the co‐planarity of both π‐systems. This angle is slightly larger in the geometry obtained in our 2D‐US exploration (24°, *Z*‐QMCH+WAT, Figure 2). According to these results, *Z*‐QMCH would then be accessible through thermal equilibrium from the more stable QMCH, whereas in the case of 6‐BIPS derivatives it requires UV‐light irradiation. Interestingly, *Z*‐QMCH was not found in our previous 2D‐US exploration in water. The electrostatic environment exerted by the G4 (polyanion, phosphate groups negatively charged) may be responsible for the relative stabilization of this positively charged *Z*‐isomer intermediate.

In summary, our results indicate that the thermal opening of QSP to form QMCH is not a proton‐assisted process neither in water nor bound to G4. As soon as QSP opens, it forms the *E*‐QMC species, which, in the presence of protons in solution (i.e., aqueous solution at physiological pH), evolves to the more stable QMCH species. In the DNA context, QSP follows the same reaction mechanism. However, the electrostatics of the environment allows the isomerization between *Z*‐QMCH and *E*‐QMCH, the latter species being the most stable one. In both cases, the pH value of the solution will control the QMC/QMCH ratio. At low pH values or physiological pH, the equilibrium will be shifted to QMCH. At high pH values, it is expected to have a representative population of QMC. The role of the DNA may be the same as the one of an excess of protons: as G4 binds more strongly to QMCH species over QSP or QMC, it will shift the equilibrium towards the QMCH:G4 complex. This chemical equilibrium is different from other SP derivatives. The quinolizidine substitution increases the thermodynamic stability of the QMC with respect to the closed QSP isomer, owing to its structural and chemical nature (i.e., tertiary amine group vs. ‐NO_2_ group in 6‐nitro BIPS), which was the result of a structure‐based G4 probe design.^6^ These features make the QSP an excellent candidate as a G4 probing system.

**Figure 3:**
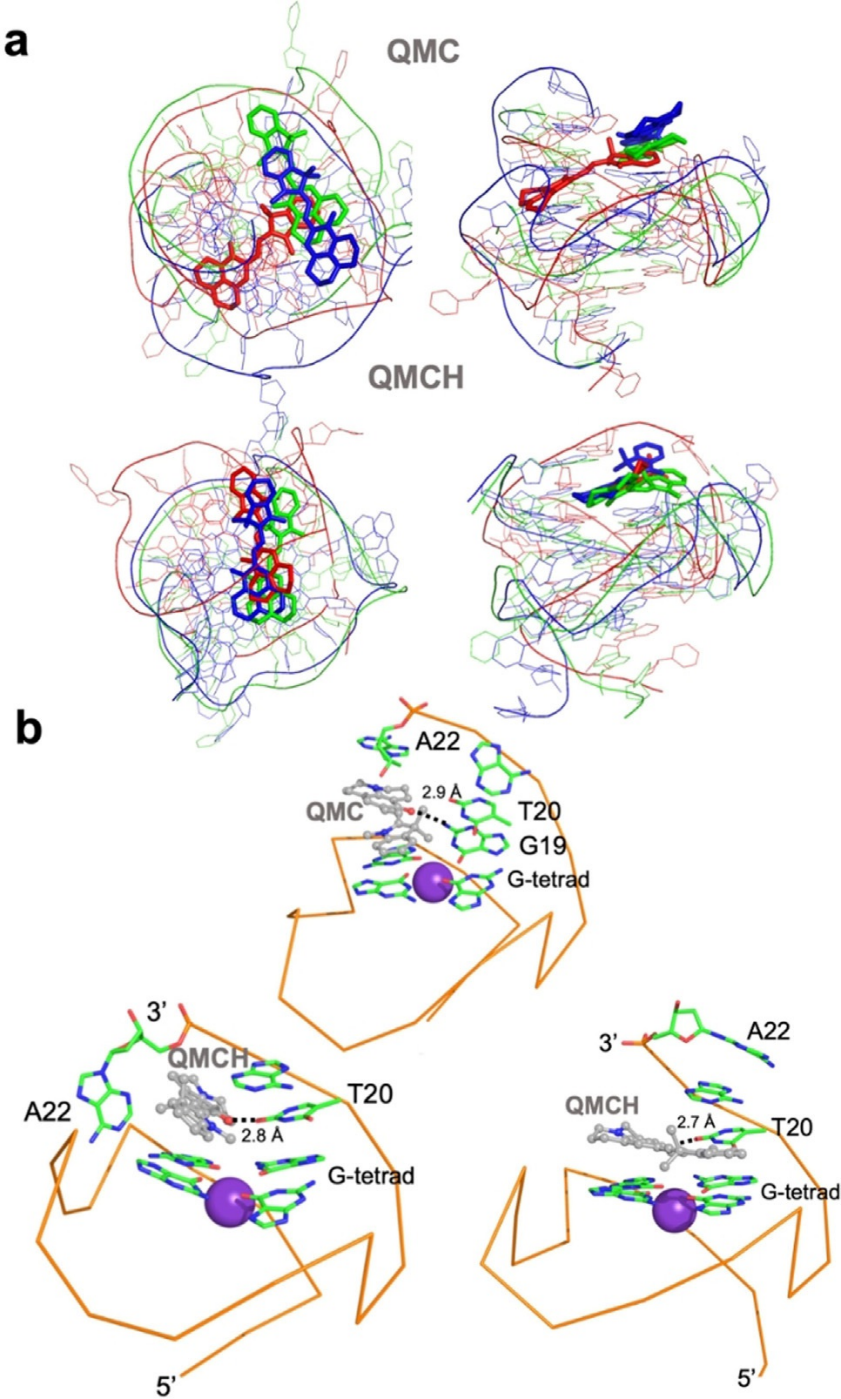
(a) Superimposition of the final geometries of the three independent fully relaxed MD simulations for both QMC and QMCH. Top and lateral views are provided. The geometries of each of the three independent MD simulation are colored in red, blue, and green, respectively, and the probe is shown as sticks. (b) Detail of the binding mode of QMC and QMCH to G4 obtained from one of the MD simulations. Only the surrounding nucleobases and the upper G‐tetrad+K^+^ ion are shown for simplicity. The backbone is shown as a ribbon. Two solutions are shown for QMCH on the bottom as an example of the mobility of A22 and show the flipping of the 3′‐terminal A22 when QMCH binds, peculiar of the QMCH:G4 complex.

To validate the former hypothesis, we performed all‐atom MD simulations, binding the three species QSP, QMC, and QMCH to the parallel‐stranded DNA G4 of the promoter c‐MYC. To this end, the three species QSP, QMC, and QMCH were positioned on the surface of the external G‐tetrads at the 3′‐end of the DNA by superimposition of each of the compounds with one of the quindoline molecules present in the solution structure of a 2:1 quindoline‐c‐MYC G4 (template structure: PDB id: 2L7V).^12^ Then, three independent 300 ns MD simulations were carried out for each system for a total of 0.9 μs each, to efficiently explore the conformational space without restraints (Section S1 in the Supporting Information). As found for other spiropyran forms when binding to DNA,^15^ the QSP species does not show a stable binding mode to G4; indeed, after a few ns, QSP moves into the bulk solvent (data not shown). In contrast, QMC and QMCH bind to G4 owing to their planar extended π system, stacking with the guanine rings of upper G‐tetrads at the 3′‐terminus. Figure 3 a shows the structural superposition of the final geometries obtained from the three independent MD trajectories for QMC:G4 and QMCH:G4 complexes. Although for QMCH all three trajectories ended up in a similar binding mode, in QMC we found more than a single solution (Figure S3 in the Supporting Information). In the most stable binding mode of QMC (Table 1, QMC simulation 2), the probe interacts through its negatively charged oxygen with the exocyclic amine group of G19 (dashed line Figure 3 b and Figure S4 in the Supporting Information). However, in the other two QMC simulations, this interaction is missing and the binding with the macromolecule is mainly through π‐stacking interactions with the surrounding guanines (simulations 1 and 3, figure not shown). In contrast, the hydroxyl group of QMCH is hydrogen bonded with the carbonyl oxygen on C‐2 of T20 (Figure 3 b). The 3′‐terminus, where A22 is located, shows high mobility along the simulation time, but the most stable complex (Table 1, simulation 1) is formed when the 3′‐terminus folds around the probe as shown in Figure 3 b. This kind of interaction has been already reported for similar G4 probes.^16^, ^17^

**Table 1:** Binding energy (kcal mol^−1^) of QMCH and QMC to c‐MYC G4 and its components along the MD simulations computed with MM‐ISMSA.^18^

	Energy components^[a, b]^					
Simulation	vdW	Electrostatics	L desolv.	R desolv.	Nonpolar	Total
**QMCH**						
1	−55.19±3.97	−8.95±0.59	−0.29±0.11	11.49±0.93	−2.54±0.11	−55.48±3.85
2	−47.77±4.30	−8.76±0.53	−0.08±0.44	10.94±0.90	−2.47±0.17	−48.14±4.50
3	−50.05±1.73	−8.63±0.49	−0.22±0.07	10.93±0.66	−2.41±0.05	−50.39±2.03
**QMC**						
1	−48.68±6.07	−0.17±0.29	3.13±0.40	11.06±1.29	−2.57±0.32	−37.24±5.45
2	−52.86±3.11	−0.73±0.15	2.34±0.22	10.06±1.01	−2.64±0.13	−43.28±2.64
3	−42.02±2.79	−9.18±0.58	−0.63±0.16	13.20±1.01	−2.31±0.09	−40.94±2.09

[a] vdW=van der Waals, L desolv.=ligand desolvation, R desolv.=receptor desolvation. [b] A window of 20 ns was analyzed for each simulation.

**Figure 4:**
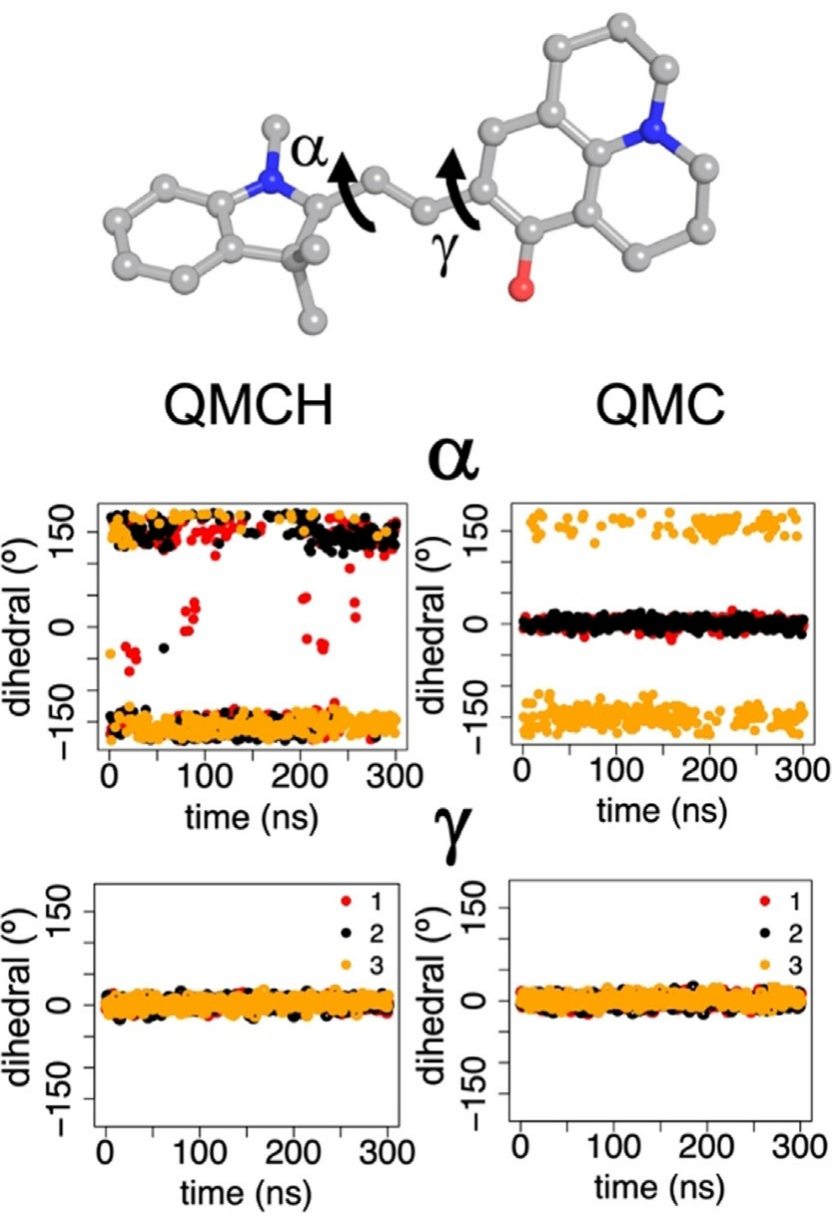
Time evolution of dihedrals α and γ (degree) along the three independent 300 ns MD simulations (red, black, and yellow points, respectively) of QMCH and QMC bound to G4. As an illustration, the two dihedrals are shown on the structure of the TTT isomer (−180°, 180°) of QMC. For each of the dihedrals, values of approximately 0° correspond to a *cis* conformation and values close to ±180° to a *trans* rotamer. In all cases, only the *trans* isomer of the β‐double bond is considered.

The indoline ring shows higher mobility than the chromene‐quindoline one along the MD simulations. This property can be measured by monitoring the evolution of the dihedral angle around the α‐bond (Figure 4) along the simulation time, as this bond connects the indoline ring to the exocyclic double bond. QMC simulations show a dihedral angle between −50 and 50 degrees around the α‐ bond, which corresponds to a *cis* conformation whereas for QMCH mostly *trans* α‐bond conformers are found. In contrast, the dihedral angle controlling the γ‐bond is fixed as *cis* in QMCH but varies to *cis* and *trans* in QMC. This means that the major conformer of QMCH is TTC and both CTC and TTC isomers dominate QMC. The hydrogen bond between the quinolizidine hydroxyl group of QMCH (absent in QMC) and G4 (see Figure 3 c) explains the ‘rigidity’ of the quinolizidine ring with a *cis* conformation of the γ‐bond, and thereby, the preference for the TTC isomer.

Finally, we analyzed the binding energies of QMC and QMCH along the three different MD simulations by using the MM‐ISMSA method^18^ (Table 1 and Section S5 in the Supporting Information).

In all the simulations, QMCH binds more strongly to G4 than QMC (Δ*E*_QMC/QMCH_≈10 kcal mol^−1^). Looking at the energy decomposition, the van der Waals component (vdW) is the largest term in both complexes. Both species present comparable values for this term, but the electrostatics make a difference, with an extra stabilization of approximately 8 kcal mol^−1^ in QMCH. In contrast to QMC, QMCH is positively charged, and as a general trend in other merocyanine species, this favors its interaction with DNA.^15^ These binding energies agree with the data obtained from the 2D free energy surface shown in Figure 2, that is, QMCH is the absolute minimum, supporting the initial hypothesis of QMCH species being the strongest binder to G4.

## Conclusion

In summary, we unraveled the molecular mechanism governing the binding of the fluorescent quinolizidine‐substituted spiropyran QSP to c‐MYC G4. We described the QSP ring opening to the QMC/QMCH open forms and characterized the dynamical binding of these merocyanine isomers to the macromolecule. The calculations show that the opening of QSP is not a proton‐assisted process, and the QMC species is an intermediate, both in solution and in G4. In the latter case, G4 would trap the QMCH form, shifting the chemical equilibrium QSP↔QMC/QMCH towards the protonated form. A similar effect can be expected at low pH value solutions. Extensive MD simulations on the μs‐scale with QMC and QMCH bound to c‐MYC G4 support that QMCH is the strongest G4 binder, showing a major rotamer around the *E*‐double β‐bond, which share a common binding site. QMC, in contrast, presents higher degrees of rotations and not a common binding mode. These results shed light on the structural features that govern the binding of QSP to G4s and open up a great opportunity to use the found structural models to design novel SP derivatives with enhanced G4 binding activity.

## Conflict of interest

The authors declare no conflict of interest.

## Supporting information

As a service to our authors and readers, this journal provides supporting information supplied by the authors. Such materials are peer reviewed and may be re‐organized for online delivery, but are not copy‐edited or typeset. Technical support issues arising from supporting information (other than missing files) should be addressed to the authors.

Supplementary
