# Supplementary material for: Spiropyran Meets Guanine Quadruplexes: Isomerization Mechanism and DNA Binding Modes of Quinolizidine‐Substituted Spiropyran Probes
Source: Chemistry. 2020 Sep 17;26(57):13039–45. doi: 10.1002/chem.202001586 (PMC7589282; doi:10.1002/chem.202001586)
Supplement: Supplementary file 1 — Supplementary [file CHEM-26-13039-s001.pdf]

Chemistry–A European Journal

Supporting Information

Spiropyran Meets Guanine Quadruplexes: Isomerization Mechanism and DNA Binding Modes of Quinolizidine-Substituted Spiropyran Probes

Davide Avagliano,[a] Pedro A. Sánchez-Murcia,*[a] and Leticia González*[a, b]

Table of contents

**Section S1**	System setup and classical molecular dynamics simulations
**Section S2**	Two-dimensional umbrella sampling (2D-US) QM/MM MD simulation
**Section S3**	Umbrella Sampling partition scheme
**Section S4**	Geometry optimization by means of Møller-Plesset second order perturbation theory calculations
**Section S5**	Binding energy calculation
**Section S6**	Cartesian coordinates of RI-MP2/def2-TZVP optimized geometries
**References**	

Section S1: System setup and classical molecular dynamics simulations

The initial cartesian coordinates of the complexes between the QSP, QMC and QMCH forms and the DNA guanine quadruplex (G4) of the promotor c-MYC were obtained by manual superimposition of the julolidine moiety of QSP with the quindoline ligand at the 3'-flank in the NMR-solved structure quindoline:DNA G4 deposited in the Protein Data Bank (PDB id. 2L7V). The force field parameters for the molecular dynamics (MD) for the three forms of the probe were generated following the general procedure described elsewhere[1] (geometry optimization and atom-centered ESP charge computed at the HF/6-31G* level) and their atom types were described as GAFF atom types. The Amber force field ff14SB[2] was used for the description of the system. The QSP:DNA G4 complex was solvated with a box of ~12500 TIP3P water molecules with a distance from the solute to the border of 20 Å. 18 Na^+ (QSP, QMC) or 17 Na^+ (QMCH) atoms were added to ensure the electroneutrality of the system. Before running the QM/MM (quantum mechanical/molecular mechanics) MD studies, the system was initially minimized in three steps where the hydrogen atoms, the solvent molecules and all the system were sequentially minimized. After that, the system was heated from 100 to 300 K in 20 ps using a time step of 0.2 fs and the weak-coupling thermostat. For this step the position of the all the heavy atoms on the ligand and DNA G4 had to be restrained (harmonic constant = 40 $kcal^{-1}$ mol^{-1} $Å^{-2}$). Finally, the restrains were gradually removed in 6 steps (6 x 20 ps) and the system switched from an NVT ensemble (steps 1-4) to an NPT ensemble (steps 5 and 6). In all the simulations, periodic boundary conditions were used, and the electrostatic interaction were treated with the Ewald method (grid space of 1 Å and cutoff distance of 10 Å). The ligand:DNA G4 complexes were further simulated for 3 x 300 ns. A random seed was placed in the heating step for the generation of the three independent simulations. The SHAKE algorithm was used to treat the bonds where hydrogen atoms were involved.

Section S2: Two-dimensional umbrella sampling (2D-US) QM/MM MD simulation

The equilibrated snapshots after heating of QSP in solution or QSP:G4 QSP obtained in Section S1 were used as initial geometries for the umbrella sampling (US) studies after a short MD minimization (see Section S1). QSP and the closest water molecule to the spiropyran oxygen were treated at the density functional tight binding method version 3 (DFTB3)[3] whereas the rest of the system (c-MYC G4, solvent molecules and counter ions) were treated with force fields. A cut off of 10 Å was defined for the electrostatic interactions between the QM and MM. Two reaction coordinates (RC) were defined for the QSP ring opening: the distance between C and O atoms of the spiro-junction of the probe (O-C bond cleavage, RC_1) and the linear combination of distances of the distances O_{water}-H_{water} and H_{water}-O_{spiro} (proton transfer, RC_2). The reaction pathway was explored by means of 2ps-steered QM/MM MD dynamics imposing a harmonic restraint (50 $kcal^{-1}$ mol^{-1} $Å^{-2}$) for both RC_1 and and RC_2. Six different reaction pathways with RC-values ($1.2 < RC_1 < 6$) Å and ($-1.2 < RC_2 < 1.2$) were defined to efficiently explore the energy surface. 50 points per trajectory along the chemical pathway were printed. Then, these snapshots were used as initial geometries for the 2D-US. Each of the windows in the 2D-US QM/MD MD study was allowed to oscillate for 5 ps around the reaction coordinate value (harmonic constant = 100 $kcal^{-1}$ mol^{-1} $Å^{-2}$ and a time step of 0.1 fs). The 2D free energy landscape was constructed using the maximum likelihood principle implemented in the variational Free Energy Profile (vFEP) method.[4]

Section S3: Umbrella Sampling partition scheme

We partitioned the (RC_1, RC_2) space in up to six sub-regions, as shown in Figure S1. Region 1 represents the exploration of all the range of both two coordinates from the respective starting (RC_1=1.2 Å, RC_2=-1.2 Å) and final (RC_1 = 6 Å, RC_2 = 1.2) values of interest. Region 2 is the opening (RC_1= 1.2:6.0 Å) with proton assistance (RC_2 = -1.2:0 Å). Region 3 is the opening (RC_1= 1.2:6.0 Å) mediated by the proton transfer (RC_2 = 0:1.2 Å). Regions 4 and 5 represent the effect of the protonation (RC_2 = -1.2:1.2 Å) in the first and the second part of the ring opening process, respectively. Finally, region 6 was explored to sample the possible protonation of the close formed before the ring opening occurs.

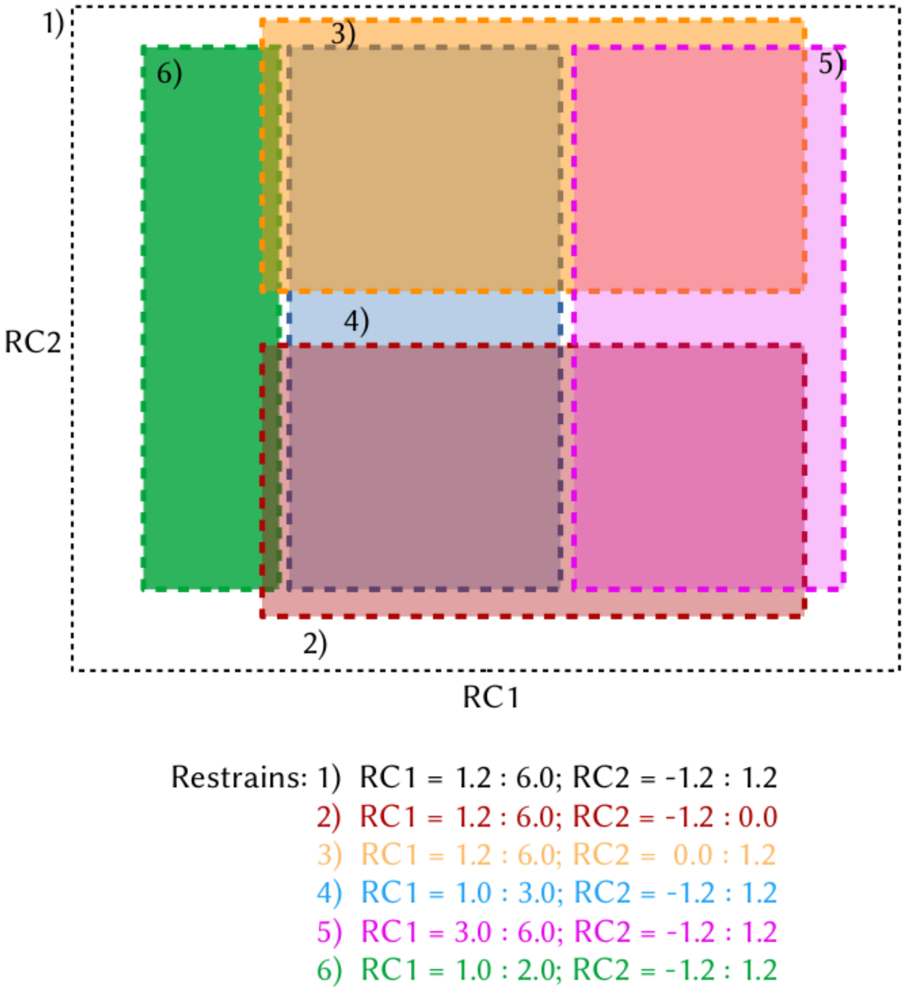

Figure S1: Schematic representation of reaction coordinates partitioning for the 2D-QM/MM-US calculation.

In total, we got 600 umbrella sampling windows (~600000 data points) that were used to calculate the free energy profile. The use of a large number of data is mandatory to get a converged surface. As example, Figure S2 shows how the FEP surface changes when the number of data increases.

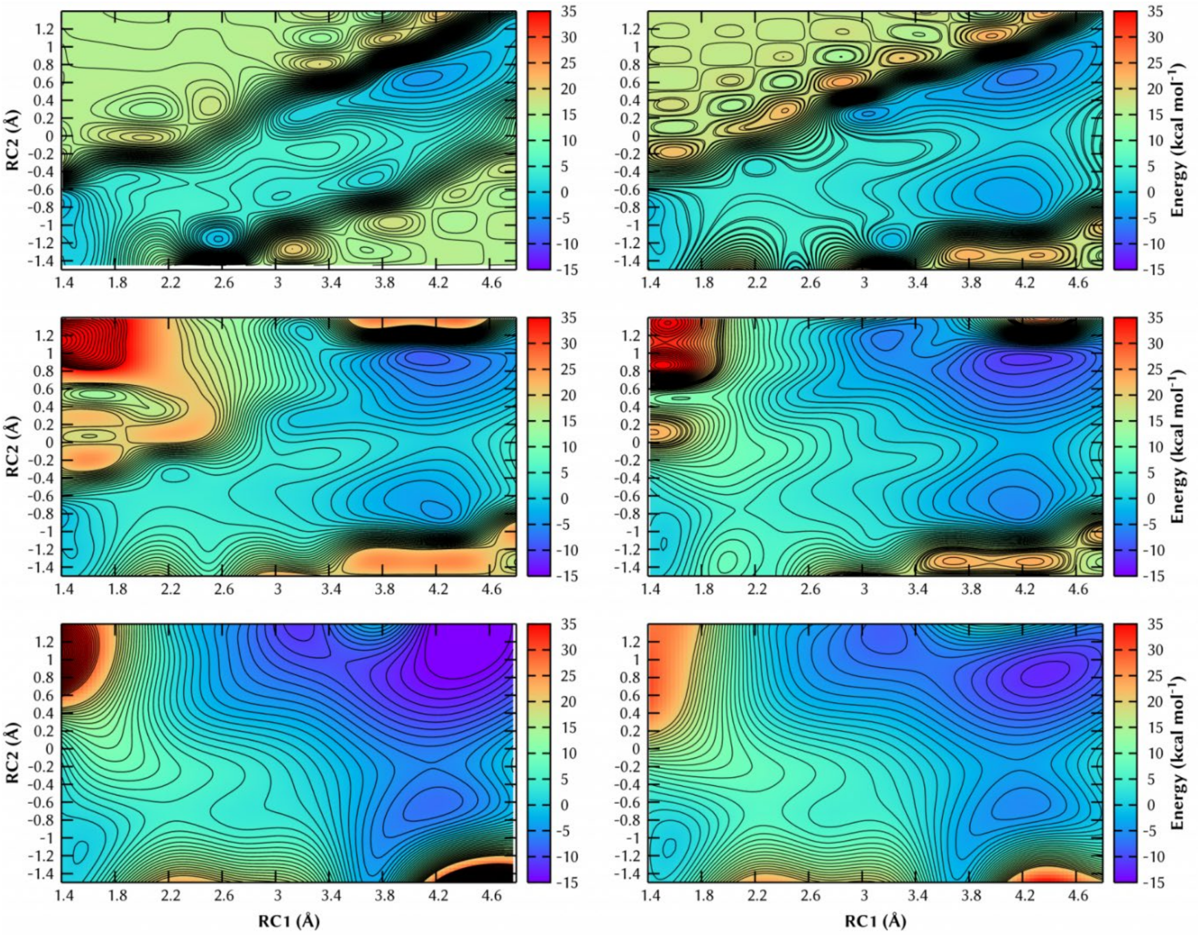

Figure S2. Free energy profiles (kcal mol^{-1}) obtained with increasing number of input data (from left to right, from top to bottom: 50, 100, 150, 200, 250 and 300 windows). The 0 of the energy is referred to the QSP minimum and each contour line represents a difference of 1 kcal mol^{-1}.

Section S4: Geometry optimization by means of Møller-Plesset second order perturbation theory calculations

Ground state geometry optimizations of the open forms of QSP in their different forms (unprotonated (QMC) and protonated (QMCH) merocyanine, and the protonated spiropyran (QSPH)) were performed with Møller-Plesset second order perturbation theory using the resolution of identity approximation (RI-MP2) and the def2-TZVP basis set. The zero-point energy correction to the Gibbs free energies at 298 K were included by means of frequency calculations. The four possible QMC isomers exocyclic double bound and the possible relative orientations of the indoline N and O atoms were taken into account in the calculations. The Z-isomers of the double bond were not considered because are known to be higher in energy. All the quantum chemical calculations were run using the TURBOMOLE suite, version 7.2.[5] The optimized geometries are reported below in section S6 in xyz format compound name//number of atom//electronic energy//atomic coordinates)

Section S5: Binding energy calculation

The binding free energies were calculated using the program MM-ISMSA.[6] For each of the simulations, the last 50 ns were considered. Before running the energy analysis, the snapshots every 10 ns were cold down in 1 ns from 300 to 100 K by means of unrestrained MD simulations. MM-ISMSA estimates solvent-corrected binding energies as well as their per-residue decomposition into van der Waals, Coulombic, apolar, and desolvation ligand/receptor contributions, using a sigmoidal, distance-dependent function for the dielectric.

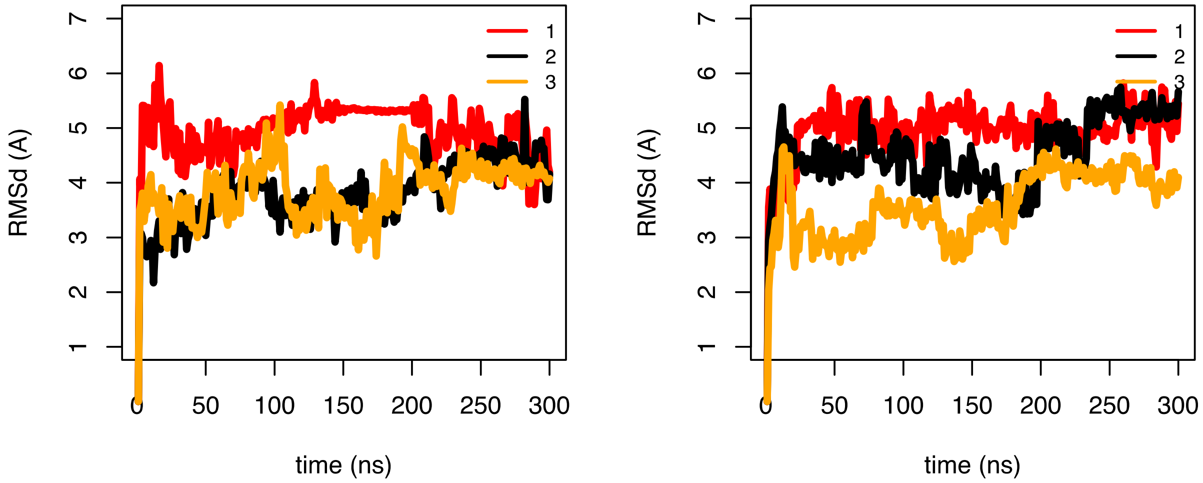

Figure S3. Root-mean squared deviation (RMSd, Å) of the complexes QMCH:G4 (left) and MC:G4 (right) long the 3 independent 300-ns MD simulations.

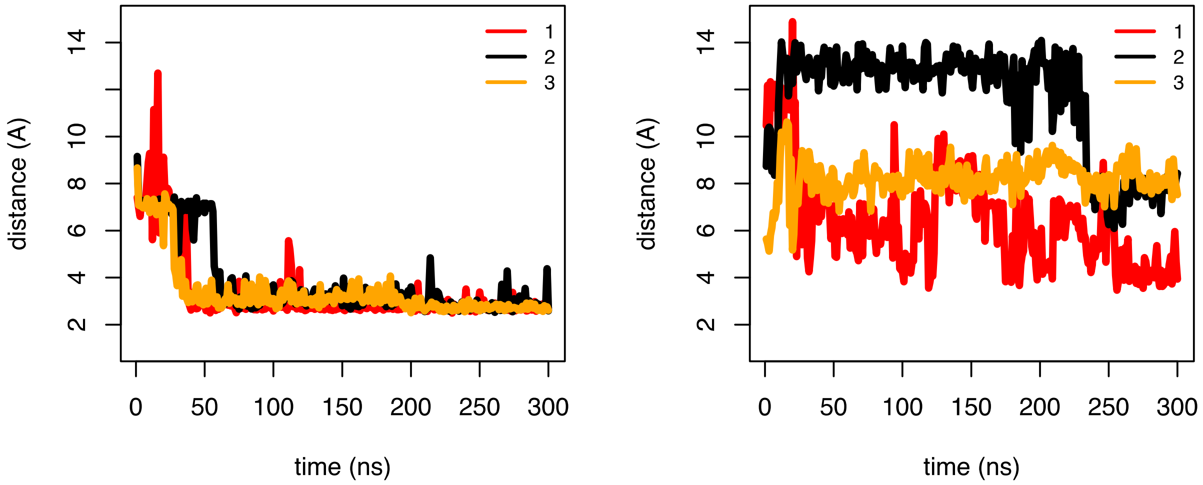

Figure S4. Distance (Å) between the spiropyran oxygen of QMCH (left) and QMC (right) and the O2 of T21 and the NH_2 group of G20, respectively, along the 3 independent 300-ns MD simulations. Whereas in QMCH the three simulations ended up in the same solution, in the case of QMC this interaction is only present in the first simulation (red line).

Section S6: Cartesian coordinates of RI-MP2/def2-TZVP optimized geometries

QSP

56

ELECTRONIC ENERGY = -1151.79461 a.u.

C	0.104629	0.890035	-0.234721
C	0.543649	1.303201	-1.496828
C	1.316771	2.464803	-1.566587
C	1.648369	3.199048	-0.435042
C	0.169483	0.497655	-2.632372
C	-0.408356	-0.707299	-2.454231
C	-0.643976	-1.247392	-1.084924
O	-0.724721	-0.185886	-0.102248
N	0.386433	-2.200901	-0.691987
C	-0.155313	-2.960512	0.349494
C	-1.553281	-2.903565	0.306134
C	-1.954668	-2.064594	-0.879901
C	0.505853	-3.693876	1.329472
C	-0.272810	-4.393882	2.258067
C	-1.665600	-4.348173	2.215210
C	-2.317246	-3.598051	1.228067
H	-3.401679	-3.552179	1.198217
H	-2.244757	-4.892097	2.952495
H	0.219913	-4.971218	3.032751
H	1.588107	-3.727150	1.382006
H	-0.650303	-1.343108	-3.296189
H	0.377928	0.866749	-3.632937
C	-3.170897	-1.181888	-0.653102
H	-3.070525	-0.588907	0.254637
H	-3.311342	-0.502677	-1.498790
H	-4.065343	-1.804973	-0.570032
C	-2.201210	-3.005370	-2.064864
H	-2.990882	-3.710745	-1.796613
H	-2.527803	-2.453074	-2.949808
H	-1.301524	-3.575014	-2.308295
C	0.418383	1.584855	0.930871
C	1.206545	2.751758	0.834021
C	-0.090332	1.059693	2.246169
C	0.642868	1.700847	3.414148
C	0.769422	3.192924	3.174689
N	1.579958	3.431638	1.994521
C	2.452103	4.467802	-0.576422
C	3.085264	4.870933	0.745672
C	2.034537	4.800053	1.836982
H	0.007694	-0.030072	2.262519
H	-1.165504	1.261174	2.322841
H	0.113948	1.511020	4.351270
H	1.648591	1.281518	3.506769
H	1.263762	3.683696	4.017032
H	-0.232676	3.643818	3.067160
H	1.195519	5.475438	1.593510
H	2.446760	5.121185	2.797127
H	3.899280	4.187249	1.002317
H	3.498995	5.880628	0.686508
H	1.795774	5.275852	-0.920595
H	3.214034	4.334160	-1.349386
C	1.755285	-1.739389	-0.573117
H	2.424403	-2.601107	-0.538274
H	1.912978	-1.131037	0.327896
H	2.008386	-1.144845	-1.451382
H	1.664125	2.808248	-2.538927

QSPH

57

ELECTRONIC ENERGY = -1152.17919 a.u.

C	-0.076271	1.166795	0.318707
C	0.318961	1.335251	-1.027784
C	1.184016	2.422972	-1.290573
C	1.674458	3.260185	-0.315789
C	-0.155197	0.597000	-2.156748
C	-0.745703	-0.636736	-2.280418
C	-0.836993	-1.695598	-1.345657
O	-0.991100	0.185401	0.596971
N	0.097482	-2.049786	-0.480270
C	-0.346563	-3.113304	0.341421
C	-1.618016	-3.491842	-0.082741
C	-2.016591	-2.631135	-1.245568
C	0.323815	-3.721176	1.393840
C	-0.346570	-4.763364	2.034749
C	-1.621076	-5.165649	1.622136
C	-2.269311	-4.537138	0.555943
H	-3.257016	-4.859894	0.244464
H	-2.112918	-5.979586	2.141542
H	0.130124	-5.269611	2.865522
H	1.309070	-3.408379	1.717990
H	-1.238627	-0.835713	-3.228592
H	-0.080926	1.150033	-3.092567
C	-3.309632	-1.851061	-0.991670
H	-3.231321	-1.248718	-0.086305
H	-3.525770	-1.188178	-1.832192
H	-4.140708	-2.550447	-0.879830
C	-2.129281	-3.469796	-2.530485
H	-2.935758	-4.196040	-2.411758
H	-2.366259	-2.837175	-3.388198
H	-1.203063	-4.012021	-2.730829
C	0.404082	1.981310	1.341205
C	1.290665	3.045093	1.038676
C	-0.037238	1.721025	2.759861
C	0.908233	2.374640	3.757250
C	1.160693	3.810562	3.348254
N	1.768561	3.851966	2.029169
C	2.574175	4.412220	-0.673388
C	3.443189	4.791051	0.513940
C	2.552841	5.039202	1.714132
H	-0.067751	0.642094	2.953265
H	-1.053555	2.105981	2.918210
H	0.486775	2.338996	4.763502
H	1.861538	1.840056	3.773549
H	1.848726	4.294694	4.043695
H	0.221898	4.383829	3.358057
H	1.889290	5.895161	1.524074
H	3.149214	5.276433	2.597967
H	4.143580	3.983484	0.744690
H	4.026513	5.688858	0.301390
H	1.962359	5.274147	-0.962773
H	3.181115	4.147867	-1.542086
C	1.436660	-1.503453	-0.389649
H	2.120565	-2.317221	-0.152646
H	1.482132	-0.734372	0.385227
H	1.698470	-1.058364	-1.348039
H	1.474419	2.603600	-2.322681
H	-1.357405	0.352850	1.478839

QMC_CTC

56

ELECTRONIC ENERGY = -1151.75953 a.u.

C	0.944291	-0.650938	-5.257444
C	1.464379	-0.136006	-3.973223
C	2.421472	0.926098	-4.002734
C	2.857117	1.505538	-5.154501
C	1.072707	-0.623555	-2.737786
C	0.093372	-1.611531	-2.502337
C	-0.304569	-2.154409	-1.304921
O	0.152668	-1.617080	-5.328777
N	0.277243	-2.061079	-0.067770
C	-0.495984	-2.713404	0.897886
C	-1.600157	-3.308847	0.283128
C	-1.548725	-3.024655	-1.192191
C	-0.276528	-2.807465	2.268330
C	-1.203278	-3.536756	3.018347
C	-2.305619	-4.143841	2.415840
C	-2.510575	-4.031783	1.036272
H	-3.368952	-4.502935	0.567570
H	-3.009427	-4.700714	3.023279
H	-1.062114	-3.625742	4.089541
H	0.564186	-2.325062	2.752689
H	-0.440715	-1.955570	-3.380305
H	1.533386	-0.129333	-1.885348
C	-2.791348	-2.268718	-1.666167
H	-2.920842	-1.343724	-1.100671
H	-2.701660	-2.018447	-2.725752
H	-3.681759	-2.887901	-1.528420
C	-1.365954	-4.317577	-1.996047
H	-2.238395	-4.963446	-1.865151
H	-1.253257	-4.091817	-3.059140
H	-0.479793	-4.859578	-1.659291
C	1.380721	0.025569	-6.455794
C	2.299890	1.061699	-6.419705
C	0.773066	-0.453829	-7.742168
C	0.946617	0.576956	-8.845169
C	2.376202	1.083138	-8.845686
N	2.680210	1.716983	-7.575578
C	3.936130	2.553528	-5.125311
C	3.839094	3.466773	-6.334680
C	3.827371	2.604808	-7.580155
H	-0.281742	-0.677416	-7.564480
H	1.228134	-1.407916	-8.033408
H	0.702394	0.152694	-9.822286
H	0.279632	1.426868	-8.674432
H	2.530653	1.827920	-9.630741
H	3.070483	0.248595	-9.036394
H	4.771261	2.037322	-7.648312
H	3.755671	3.221850	-8.479864
H	2.917195	4.054258	-6.297395
H	4.683041	4.160270	-6.371786
H	4.918377	2.063525	-5.140320
H	3.876771	3.114579	-4.189050
C	1.594927	-1.540491	0.213958
H	1.996615	-2.059408	1.084520
H	1.579306	-0.466595	0.418190
H	2.243047	-1.733165	-0.641733
H	2.829799	1.271647	-3.053854

QMC_CTT

56

ELECTRONIC ENERGY = -1151.75881 a.u.

C	1.702926	-2.490082	-0.434958
C	1.029066	-1.182978	-0.226862
C	1.827132	-0.013960	-0.042091
C	3.187458	-0.052641	-0.056046
C	-0.346894	-1.195466	-0.220930
C	-1.204801	-0.091246	-0.006800
C	-2.576313	-0.067498	-0.036754
O	1.058134	-3.545160	-0.577652
N	-3.447779	-1.046309	-0.443879
C	-4.771636	-0.654120	-0.228300
C	-4.796514	0.646729	0.279994
C	-3.383716	1.144282	0.415215
C	-5.940891	-1.373837	-0.454159
C	-7.152813	-0.740293	-0.166044
C	-7.190484	0.562203	0.332110
C	-6.003075	1.266533	0.559512
H	-6.030681	2.279104	0.950230
H	-8.145151	1.026745	0.549230
H	-8.079983	-1.277992	-0.329061
H	-5.929203	-2.392831	-0.821764
H	-0.753408	0.859538	0.258860
H	-0.763642	-2.187968	-0.352031
C	-3.061536	1.515167	1.863580
H	-3.232358	0.665158	2.526858
H	-2.017587	1.823095	1.959609
H	-3.696507	2.343434	2.189150
C	-3.136347	2.335374	-0.516737
H	-3.773286	3.175533	-0.227150
H	-2.093331	2.656929	-0.460791
H	-3.362619	2.069548	-1.551279
C	3.147242	-2.483059	-0.485998
C	3.869275	-1.315432	-0.313611
C	3.811196	-3.798527	-0.770324
C	5.236871	-3.594310	-1.254806
C	5.935705	-2.586927	-0.361684
N	5.246728	-1.309730	-0.413875
C	3.986599	1.184884	0.247712
C	5.353835	1.126554	-0.409703
C	6.025710	-0.162832	0.013191
H	3.209691	-4.336683	-1.506956
H	3.797510	-4.422618	0.130994
H	5.791555	-4.535865	-1.256886
H	5.234716	-3.206220	-2.277385
H	6.965392	-2.418868	-0.687464
H	5.970460	-2.962918	0.673555
H	6.171166	-0.164368	1.106882
H	7.014467	-0.254882	-0.444153
H	5.255830	1.140442	-1.499032
H	5.969098	1.979533	-0.112564
H	4.129187	1.264325	1.333439
H	3.423574	2.068672	-0.063224
C	-3.101148	-2.264052	-1.143837
H	-3.961127	-2.580498	-1.733128
H	-2.821831	-3.068375	-0.458964
H	-2.266574	-2.069402	-1.817845
H	1.343711	0.942168	0.142604

QMC_TTC

56

ELECTRONIC ENERGY = -1151.76334 a.u.

C	1.873780	-1.556998	-0.617503
C	1.261269	-0.282917	-0.185501
C	2.118649	0.830471	0.079525
C	3.471552	0.779906	-0.058468
C	-0.101485	-0.105872	-0.016283
C	-1.101620	-1.077401	-0.215045
C	-2.449883	-0.870914	-0.040525
O	1.205112	-2.592601	-0.835296
N	-3.357409	-1.872918	-0.260131
C	-4.661862	-1.446519	-0.016687
C	-4.632423	-0.105878	0.377029
C	-3.203969	0.378790	0.393593
C	-5.856595	-2.152706	-0.117939
C	-7.036494	-1.470428	0.188379
C	-7.019722	-0.131766	0.581630
C	-5.808335	0.561151	0.678449
H	-5.794415	1.602588	0.984840
H	-7.950967	0.371686	0.813178
H	-7.982116	-1.995927	0.118097
H	-5.889889	-3.192411	-0.420341
H	-0.751122	-2.052908	-0.526863
H	-0.400353	0.891397	0.298508
C	-2.818220	0.811056	1.812991
H	-2.956664	-0.014923	2.513640
H	-1.777371	1.132469	1.864229
H	-3.454249	1.641756	2.130298
C	-3.031781	1.523213	-0.612084
H	-3.669913	2.363601	-0.326034
H	-1.998511	1.869982	-0.650281
H	-3.321097	1.197229	-1.613270
C	3.305925	-1.562888	-0.798691
C	4.083849	-0.446288	-0.536804
C	3.900317	-2.843667	-1.309185
C	5.289489	-2.612460	-1.880117
C	6.093276	-1.751232	-0.925114
N	5.448331	-0.461951	-0.755743
C	4.326324	1.955823	0.329032
C	5.639439	1.947304	-0.433583
C	6.294037	0.595752	-0.235752
H	3.224200	-3.264391	-2.057490
H	3.936354	-3.582939	-0.500147
H	5.807024	-3.559607	-2.051918
H	5.220203	-2.091424	-2.839328
H	7.099559	-1.568087	-1.310747
H	6.196112	-2.262013	0.046187
H	6.512941	0.442000	0.834765
H	7.245780	0.542092	-0.771187
H	5.462807	2.106486	-1.501251
H	6.305554	2.737323	-0.077592
H	4.549729	1.904156	1.402579
H	3.770080	2.882573	0.166071
C	-2.974791	-3.199478	-0.684332
H	-3.867566	-3.805994	-0.813198
H	-2.325567	-3.665957	0.060927
H	-2.431789	-3.152446	-1.631448
H	1.660486	1.754099	0.430932

QMC_TTT

56

ELECTRONIC ENERGY = -1151.76134 a.u

C	1.975316	-2.027077	-5.432329
C	2.161133	-1.117652	-4.273006
C	2.795915	0.144740	-4.477595
C	3.213629	0.557556	-5.705139
C	1.704701	-1.570369	-3.057718
C	1.749123	-0.891432	-1.818778
C	1.276419	-1.414754	-0.641851
O	1.475660	-3.159514	-5.304492
N	1.335818	-0.730367	0.547571
C	0.789042	-1.473230	1.593335
C	0.349626	-2.701029	1.092467
C	0.625462	-2.769927	-0.389467
C	0.655996	-1.141063	2.937911
C	0.063797	-2.085862	3.779798
C	-0.378322	-3.315766	3.291866
C	-0.236262	-3.630202	1.936219
H	-0.580265	-4.586810	1.555370
H	-0.834185	-4.029492	3.967925
H	-0.052599	-1.853843	4.832387
H	0.991643	-0.191070	3.336235
H	2.175700	0.105524	-1.789075
H	1.269613	-2.562673	-3.104170
C	-0.695165	-2.924122	-1.154649
H	-1.363885	-2.089578	-0.933144
H	-0.534603	-2.963862	-2.232494
H	-1.188105	-3.849812	-0.846286
C	1.583799	-3.930649	-0.685120
H	1.123835	-4.871338	-0.371185
H	1.816182	-4.000376	-1.748323
H	2.517359	-3.804549	-0.132575
C	2.381982	-1.529856	-6.727208
C	2.969581	-0.285505	-6.869772
C	2.105586	-2.422131	-7.901615
C	2.131395	-1.632833	-9.199741
C	3.355255	-0.737162	-9.223290
N	3.310355	0.202077	-8.116512
C	3.973655	1.846390	-5.857211
C	3.782218	2.431096	-7.245211
C	4.157550	1.371052	-8.259170
H	1.139655	-2.908691	-7.745752
H	2.841230	-3.234507	-7.931295
H	2.142483	-2.298471	-10.066343
H	1.240433	-1.002828	-9.274444
H	3.399645	-0.154746	-10.147169
H	4.270047	-1.349335	-9.170753
H	5.222814	1.107756	-8.143687
H	4.025866	1.743528	-9.278599
H	2.738736	2.721518	-7.397618
H	4.406288	3.316912	-7.387793
H	5.044345	1.653619	-5.708914
H	3.666512	2.546098	-5.075619
C	1.897581	0.590346	0.671381
H	1.849756	0.906317	1.710407
H	1.340797	1.305039	0.058599
H	2.942979	0.592859	0.350179
H	2.988877	0.792054	-3.625540

QMCH_CTC

57

ELECTRONIC ENERGY = -1152.18104 a.u.

C	1.863283	0.903793	-0.255247
C	1.189884	-0.328983	-0.061439
C	2.005156	-1.466537	0.152933
C	3.376678	-1.424780	0.166777
C	-0.212095	-0.522322	-0.069566
C	-1.219668	0.416387	-0.154068
C	-2.599890	0.150718	-0.114993
O	1.091884	2.001724	-0.494088
N	-3.237525	-0.979746	-0.422069
C	-4.630393	-0.856961	-0.194079
C	-4.896305	0.441363	0.232361
C	-3.606126	1.203950	0.297317
C	-5.614870	-1.822763	-0.352991
C	-6.923385	-1.430168	-0.068134
C	-7.213700	-0.129212	0.352377
C	-6.201483	0.821402	0.506757
H	-6.436840	1.827812	0.836339
H	-8.240631	0.142434	0.565894
H	-7.726535	-2.149610	-0.173053
H	-5.396592	-2.836856	-0.664708
H	-0.955342	1.464111	-0.157043
H	-0.500936	-1.559179	0.081918
C	-3.308651	1.705527	1.714529
H	-3.309061	0.880940	2.429948
H	-2.336142	2.201038	1.750981
H	-4.074233	2.423807	2.015220
C	-3.596008	2.364551	-0.708700
H	-4.376300	3.079275	-0.438978
H	-2.636161	2.884938	-0.693740
H	-3.790231	2.008153	-1.722177
C	3.246335	1.000760	-0.211002
C	4.029646	-0.169783	-0.015657
C	3.901912	2.348454	-0.376606
C	5.326402	2.333202	0.156333
C	6.058017	1.131067	-0.401613
N	5.385096	-0.094518	0.000586
C	4.187996	-2.677740	0.350757
C	5.536651	-2.341652	0.963668
C	6.213464	-1.290434	0.108676
H	3.336081	3.114189	0.167108
H	3.908460	2.639283	-1.434614
H	5.848666	3.250808	-0.120156
H	5.316736	2.267757	1.247237
H	7.078903	1.086446	-0.018776
H	6.117080	1.189902	-1.497586
H	6.427180	-1.691977	-0.891622
H	7.164732	-0.984808	0.549590
H	5.405715	-1.954533	1.977954
H	6.173796	-3.226034	1.022327
H	4.343671	-3.159362	-0.621374
H	3.633682	-3.384864	0.971842
C	-2.667542	-2.166944	-1.036838
H	-3.417041	-2.600077	-1.697164
H	-2.383958	-2.902392	-0.282297
H	-1.794288	-1.876356	-1.619458
H	1.670522	2.762485	-0.651452
H	1.513308	-2.424685	0.303860

QMCH_CTT

57

ELECTRONIC ENERGY = -1152.18103 a.u.

C	1.714737	-2.379056	-0.406075
C	1.025814	-1.143904	-0.292951
C	1.815722	0.022510	-0.185532
C	3.187227	-0.009528	-0.161931
C	-0.386758	-1.143417	-0.292095
C	-1.202713	-0.043461	-0.130738
C	-2.606534	-0.033656	-0.103451
O	0.939452	-3.494582	-0.507655
N	-3.451463	-0.955929	-0.570020
C	-4.794680	-0.602398	-0.292239
C	-4.805150	0.638162	0.339414
C	-3.391242	1.114288	0.497918
C	-5.949333	-1.319574	-0.575172
C	-7.157434	-0.729161	-0.201567
C	-7.190337	0.520203	0.423753
C	-6.011902	1.217581	0.701223
H	-6.046968	2.185150	1.190648
H	-8.145408	0.950020	0.701297
H	-8.085143	-1.252823	-0.398609
H	-5.933756	-2.294856	-1.045875
H	-0.750622	0.919280	0.079773
H	-0.841422	-2.122788	-0.371843
C	-3.020534	1.305078	1.972738
H	-3.187402	0.387484	2.540049
H	-1.972244	1.594018	2.074693
H	-3.638362	2.095190	2.404592
C	-3.147683	2.402602	-0.300936
H	-3.780754	3.197851	0.098105
H	-2.107308	2.724281	-0.218473
H	-3.392785	2.263537	-1.355629
C	3.098710	-2.456340	-0.426543
C	3.860839	-1.266319	-0.282538
C	3.773629	-3.792631	-0.602419
C	5.213352	-3.619135	-1.060706
C	5.900781	-2.598751	-0.177862
N	5.215748	-1.317109	-0.265362
C	3.985090	1.253361	0.009779
C	5.351850	1.101299	-0.634930
C	6.029035	-0.123583	-0.056936
H	3.238153	-4.390235	-1.349029
H	3.752932	-4.354975	0.339347
H	5.747420	-4.569966	-1.017969
H	5.237171	-3.266844	-2.095035
H	6.932664	-2.442080	-0.495870
H	5.923479	-2.943940	0.865046
H	6.221035	0.016657	1.015677
H	6.991330	-0.300678	-0.542009
H	5.249108	0.983218	-1.717217
H	5.974058	1.979144	-0.451223
H	4.112498	1.462011	1.078352
H	3.434122	2.094798	-0.416290
C	-3.115420	-2.127684	-1.360855
H	-3.954234	-2.345644	-2.018942
H	-2.922186	-2.991075	-0.721968
H	-2.232786	-1.908315	-1.960741
H	1.507832	-4.277546	-0.518773
H	1.329776	0.990260	-0.105394

QMCH_TTC

57

ELECTRONIC ENERGY = -1152.18407 a.u.

C	1.962457	-1.569754	-0.658688
C	1.269662	-0.400011	-0.253345
C	2.064435	0.740486	0.020511
C	3.433111	0.750295	-0.067857
C	-0.128558	-0.268108	-0.095865
C	-1.122521	-1.206221	-0.283326
C	-2.485816	-0.934807	-0.086032
O	1.214029	-2.680983	-0.911906
N	-3.417153	-1.870031	-0.303273
C	-4.713391	-1.398127	-0.003805
C	-4.606576	-0.072487	0.406926
C	-3.159889	0.342236	0.384601
C	-5.926055	-2.071499	-0.077148
C	-7.065207	-1.348735	0.277846
C	-6.979031	-0.014869	0.686068
C	-5.745720	0.637314	0.755568
H	-5.688518	1.671991	1.076747
H	-7.883244	0.518133	0.954805
H	-8.033022	-1.833794	0.236595
H	-6.006544	-3.106822	-0.384025
H	-0.842401	-2.203441	-0.586365
H	-0.432678	0.726587	0.213414
C	-2.714157	0.733312	1.800924
H	-2.863434	-0.095994	2.495281
H	-1.664387	1.024967	1.832179
H	-3.315242	1.578087	2.143906
C	-2.968023	1.488607	-0.619784
H	-3.573003	2.342065	-0.306364
H	-1.928074	1.809727	-0.679046
H	-3.294448	1.186612	-1.617003
C	3.341503	-1.594518	-0.803148
C	4.104757	-0.436359	-0.488132
C	4.015232	-2.852686	-1.289714
C	5.410819	-2.557580	-1.817511
C	6.159732	-1.712179	-0.809506
N	5.457331	-0.456843	-0.590285
C	4.224341	1.978279	0.289102
C	5.541233	1.988943	-0.467877
C	6.270658	0.689384	-0.197595
H	3.428881	-3.308533	-2.096390
H	4.077448	-3.587806	-0.477445
H	5.953813	-3.485636	-2.004759
H	5.345671	-2.010145	-2.761183
H	7.159854	-1.470310	-1.172632
H	6.273434	-2.253742	0.140012
H	6.535915	0.614467	0.865968
H	7.198251	0.637877	-0.771688
H	5.359939	2.086459	-1.541859
H	6.167676	2.826889	-0.156569
H	4.427086	1.982720	1.366166
H	3.632922	2.870537	0.072304
C	-3.141997	-3.219122	-0.771971
H	-4.073921	-3.674050	-1.094547
H	-2.703004	-3.815606	0.029518
H	-2.457332	-3.180189	-1.618313
H	1.557404	1.649874	0.334448
H	1.808547	-3.410861	-1.140286

QMCH_TTT

57

ELECTRONIC ENERGY = -1152.17833 a.u.

C	1.736422	-1.857495	-5.534949
C	1.939353	-1.086344	-4.353393
C	2.668238	0.122313	-4.499258
C	3.220930	0.516240	-5.687970
C	1.492264	-1.554425	-3.104206
C	1.532169	-0.872002	-1.900911
C	1.140184	-1.418542	-0.674429
O	1.020102	-3.017881	-5.526563
N	1.176942	-0.703995	0.459787
C	0.782173	-1.472946	1.574670
C	0.451474	-2.748936	1.126825
C	0.644466	-2.821730	-0.364620
C	0.711068	-1.101554	2.911303
C	0.278308	-2.076407	3.810671
C	-0.063549	-3.360599	3.378787
C	0.020378	-3.709785	2.028796
H	-0.244190	-4.710181	1.702732
H	-0.395653	-4.094687	4.103118
H	0.208729	-1.830100	4.863375
H	0.978865	-0.112815	3.262365
H	1.893262	0.149369	-1.895408
H	1.105329	-2.567066	-3.070411
C	-0.705655	-3.108966	-1.038135
H	-1.435954	-2.337292	-0.786780
H	-0.618349	-3.160372	-2.123765
H	-1.086119	-4.069012	-0.683112
C	1.695734	-3.888488	-0.700161
H	1.347494	-4.859890	-0.342683
H	1.874877	-3.964174	-1.773186
H	2.643961	-3.663788	-0.207796
C	2.265747	-1.491039	-6.758339
C	3.031133	-0.297735	-6.854601
C	2.002639	-2.345848	-7.967552
C	2.192475	-1.542087	-9.242096
C	3.523016	-0.820600	-9.188437
N	3.569310	0.074404	-8.035211
C	4.055357	1.762068	-5.780679
C	3.958480	2.344146	-7.180305
C	4.375024	1.284770	-8.178656
H	0.993304	-2.755127	-7.905238
H	2.682941	-3.204826	-7.961502
H	2.163114	-2.193284	-10.117511
H	1.392129	-0.804753	-9.348711
H	3.677380	-0.210022	-10.079212
H	4.350531	-1.539528	-9.129775
H	5.437308	1.036497	-8.053565
H	4.240173	1.641399	-9.201841
H	2.931998	2.658678	-7.388403
H	4.604969	3.216607	-7.291451
H	5.101946	1.517944	-5.563859
H	3.730137	2.479856	-5.024476
C	1.592765	0.685506	0.545110
H	1.346785	1.066192	1.531898
H	1.059979	1.276132	-0.200128
H	2.669026	0.770559	0.383603
H	2.852149	0.740883	-3.626349
H	0.416137	-3.019672	-4.771944

References

[1] D. Avagliano, P. A. Sánchez-Murcia, L. González, *Phys. Chem. Chem. Phys.* **2019**, *21*, 8614–8618.
[2] J. W. Ponder, D. A. Case, *Adv. Prot. Chem.* **2003**, *66*, 27–85.
[3] M. Gaus, Q. Cui, M. Elstner, *J. Chem. Theory Comput.* **2011**, *7*, 931–948.
[4] T. S. Lee, B. K. Radak, M. Huang, K. Y. Wong, D. M. York, *J. Chem. Theory Comput.* **2014**, *10*, 24–34.
[5] TURBOMOLE GmbH, "TURBOMOLE V7.0 2015, a development of University of Karlsruhe and Forschungszentrum Karlsruhe GmbH, 1989-2007, TURBOMOLE GmbH, since 2007; available from http://www.turbomole.com," can be found under http://www.turbomole.com
[6] J. Klett, A. Núñez-Salgado, H. G. Dos Santos, Á. Cortés-Cabrera, A. Perona, R. Gil-Redondo, D. Abia, F. Gago, A. Morreale, *J. Chem. Theory Comput.* **2012**, *8*, 3395–3408.
